# Navigating the Generosity of Medicines (Gen-MED): characterizing the complex relationships of medication donations in Japan through stakeholder and social network analysis

**DOI:** 10.3389/fpubh.2026.1866926

**Published:** 2026-08-24

**Authors:** Naoko Arakawa, Carol Namata, Eiko Kobayashi, Kayoko Takeda Mamiya, Tetsumi Irie, Takashi Egawa, Kaitlyn E. Watson

**Affiliations:** 1 Division of Pharmacy Practice and Policy, School of Pharmacy, University of Nottingham, Nottingham, United Kingdom; 2 Faculty of Pharmacy and Pharmaceutical Sciences, College of Health Sciences, University of Alberta, Edmonton, AB, Canada; 3 Department of Pharmacy, International Medical Relief, Japanese Red Cross Medical Center, Tokyo, Japan; 4 Department of Pharmaceutical Education, Faculty of Pharmaceutical Sciences, Hokkaido University of Science, Hokkaido, Japan; 5 Department of Pharmaceutical Packaging Technology, Faculty of Life Sciences, Kumamoto University, Kumamoto, Japan; 6 Department of Pharmaceutical Sciences for Health Crisis Management, Faculty of Pharmaceutical Sciences, Fukuoka University, Fukuoka, Japan; 7 Women and Children’s Health Research Institute, University of Alberta, Edmonton, AB, Canada; 8 Epidemiology Coordinating and Research (EPICORE) Centre, University of Alberta, Edmonton, AB, Canada

**Keywords:** disaster, donated medicine, donation, emergencies, humanitarian, medicine, social network analysis, stakeholder analysis

## Abstract

**Background:**

Anthropogenic and natural hazards increase reliance on donated medicines. However, inappropriate donations (e.g., expired, unlabeled, or poorly matched to local needs) remain common and create negative impacts on disaster-affected communities. In Japan, the coordination mechanisms and roles among stakeholders remain insufficiently characterized. This study aimed to identify and characterize stakeholders involved in the donated medicine cycle in Japan and to examine their relationships. In addition, this study also obtained stakeholder opinions on our proposed collaborative initiative, to build transparency within the donated medicine systems and increase the proportion of donated medicines that meet the health needs of disaster affected populations while complying with the World Health Organization (WHO) guidelines.

**Method:**

This study applied stakeholder analysis combined with social network analysis to examine the existing collaboration network within the Japanese donated medicine system. The analysis followed three phases: stakeholder identification, stakeholder categorization, and analysis of stakeholder relationships. Between June and August 2025, an online questionnaire was distributed to 18 stakeholders asking for their attributes, interests, desire for involvement, concerns, and the level of collaboration they had with other stakeholders. Data analysis combined descriptive quantitative analysis and social network analysis.

**Results:**

The analysis identified 18 stakeholders across nine groups, of which 15 organizations responded and participated in the study. Eight stakeholders were considered critical to the proposed initiative. These belonged to seven groups including pharmacy professional bodies, national and regional government, humanitarian organizations, pharmaceutical companies, regional hospitals, and the national pharmaceutical manufacturers association. A collaboration network existed between 17 stakeholders. National-level stakeholders emerged as the most strategically positioned for controlling the flow of information and resources within the Japanese donated medicine system. Two of the eight critical stakeholders were pharmacy professional bodies, highlighting their important role in Japanese donated medicine system.

**Conclusion:**

This study revealed collaboration patterns and highlighted contextual insights required for the successful implementation of the proposed initiative. These insights provide practical guidance for building transparency, and better alignment of donated medicines with local health needs.

## Graphical abstract

**Figure:**
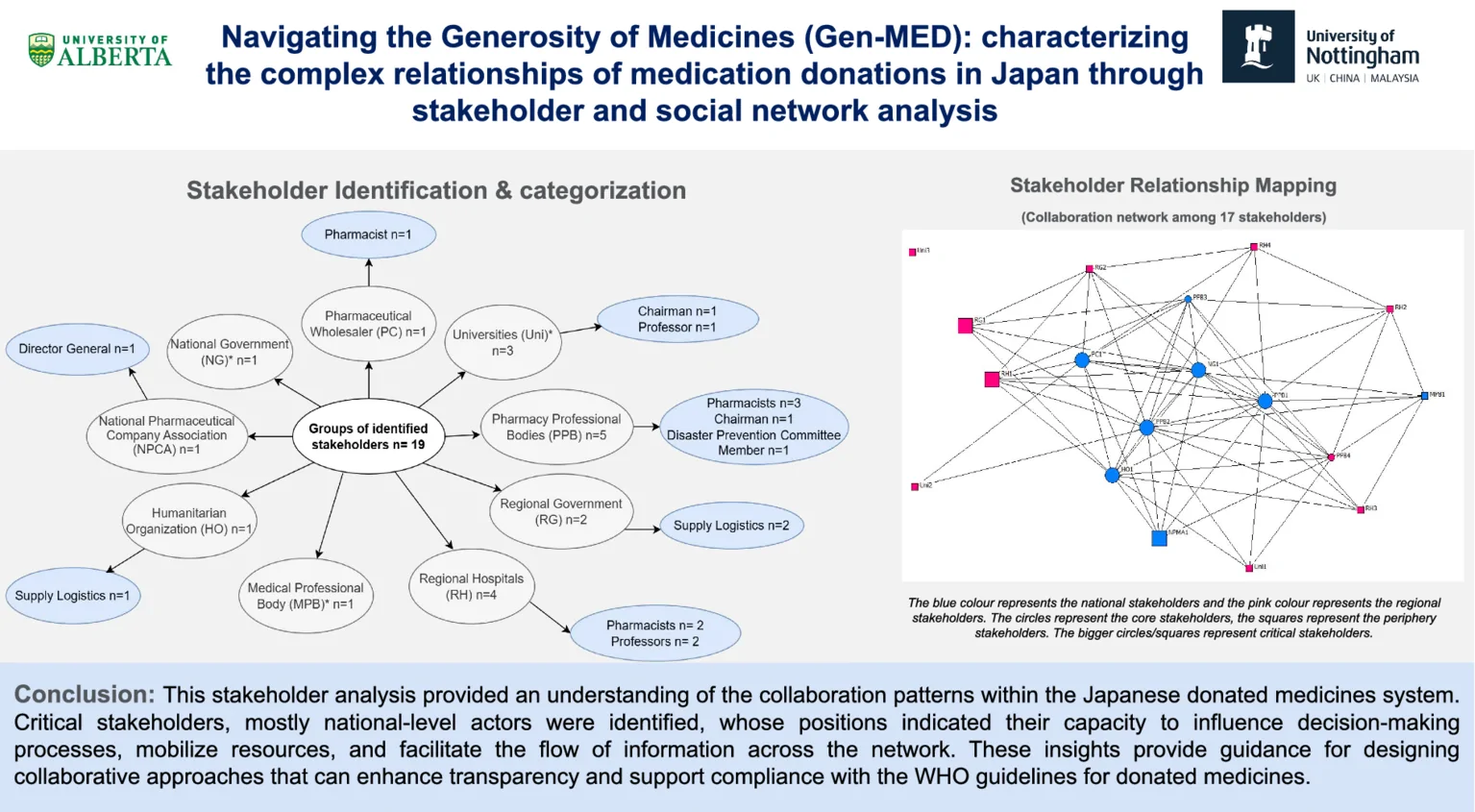

## 1 Introduction

Anthropogenic and natural hazards frequently driven by climate change can lead to profound disruptions in local health systems across both Resource-Limited Countries (RLCs) and high-income settings (1). Under these circumstances, donated medicines emerge as a fundamental resource for providing the necessary medicines to alleviate suffering and prevent morbidity and mortality (2, 3). However, the misconception that any donation is a good donation (4), including expired medicines (5) can cause significant harm. Inappropriate donated medicines (i.e., expired, improperly labeled, or poorly aligned with local health needs) can create substantial environmental, economic, social, and health burdens for recipient countries (5–7). Despite the established World Health Organization (WHO) guidelines for donated medicines (8), compliance remains a persistent global challenge (9), leading to the rise of inappropriate donated medicines (10). In Japan, the Pharmaceuticals and Medical Devices Act and Customs Act are aligned with the WHO Donated Medicines guidelines. These Acts address the necessary quality of medicines and the control of importing medicines in Japan, though there is no exact statements of the WHO guidelines alignment. During large-scale disasters in Japan, the regulations governing the donation (provision) of pharmaceuticals to disaster-affected areas by domestic corporations, medical institutions, and individuals offer greater procedural flexibility compared to overseas donations. However, they remain subject to strict quality control and distribution channel regulations based on the PMD Act (Pharmaceuticals and Medical Devices Act). The MHLW Disaster Supply Manual: Under the Ministry of Health, Labour and Welfare (MHLW) emergency guidelines, the government deploys a hybrid “Push-type” (immediate proactive delivery) and “Pull-type” (demand-driven) supply model via official industry associations, such as the Japan Pharmaceutical Manufacturers Association (JPMA).

Addressing these challenges requires a clear understanding of the stakeholders involved in donated medicines systems and the ways in which they interact. Japan provides an informative context for examining these collaborative patterns (11). Having experienced multiple large-scale natural disasters, including the Great Hanshin-Awaji Earthquake (1995), the Great East Japan Earthquake (2011), and the most recent 2024 Noto Peninsula Earthquake, all of which placed significant strain on national and local health systems (12–14), awareness of inappropriate donated medicines increased in Japan (15), and efforts have been made to improve donation practices. Evidence shows that Japan deliberately reduced reliance on unsolicited donated medicines and prioritized structured supply chains (16). To manage this, Japan developed a National Disaster Medical System (NDMS) coordinated by the Ministry of Health, Labour and Welfare (MHLW). This system integrates Disaster Medical Assistance Teams (DMAT) and the Emergency Medical Information System (EMIS) to share real-time information on medical needs and pharmaceutical availability, allowing personnel involved in disaster medical activities to adjust the distribution of medicines and services in response to fluctuating demand (17). To maintain quality control and clear lines of responsibility under the PMD Act even during disasters, domestic pharmaceutical donations and relief in Japan are strictly categorized as follows:

Negative: General Individuals and Corporations (Non-medical): Direct donation of physical medicines is strictly prohibited. As a rule, support must be provided in the form of monetary contributions, such as disaster relief funds.
Affirmative: Medical Institutions and Pharmacies: Emergency direct inter-facility mutual aid (donations or loans) is permitted under special administrative notices issued by the Ministry of Health, Labour and Welfare (MHLW). Pharmaceutical Manufacturers and Wholesalers: Donations are permitted exclusively through official distribution channels and centralized collection centers managed by local governments or pharmaceutical associations.

However, stakeholder roles and coordination mechanisms within this system remain insufficiently characterized. Evidence from previous disasters revealed how large volumes of pharmaceuticals and distribution delays resulted in significant surpluses in affected zones (16). While existing research has primarily focused on disaster response logistics and pharmaceutical supply chains, it has largely overlooked the inter-organizational relationships and collaboration structures (18–20). Improving the governance and effectiveness of donated medicines systems requires meaningful engagement with these stakeholders throughout the process of planning, co-creating, and implementing initiatives designed to reduce the environmental, economic, social, and health impacts associated with inappropriate donations (21–23). The present study is part of a broader program of work that adopts an implementation approach to the complex challenges of donated medicines. It focuses on understanding the problem, context, and stakeholders involved, to inform the co-creation of solutions and implementation strategies that enhance adoption of WHO guidelines. The Navigating the Generosity of Medicines (Gen-MED) project aims to ultimately build transparency within the donated medicines systems and increase the proportion of donated medicines that meet the health needs of affected populations while complying with WHO guidelines.

By analyzing the Japanese context, where domestically donated medicines are utilized within domestic systems, this study maps the donated medicines supply system during disasters. Although focused on a single country, it may offer insights that reflect a microcosm of global practices. An essential first step in developing collaborative initiatives is the identification of stakeholders whose interests, needs, and resources influence the system under consideration (24). Stakeholder analysis provides a systematic approach to generating knowledge about these stakeholders, their intentions, relationships, and interests, as well as the influence and resources they contribute to decision-making and implementation processes (25). To move beyond simply identifying stakeholders, this study combines stakeholder analysis with social network analysis, to map the existing collaboration network within the Japanese donated medicines system (23). This study aims to identify and categorize stakeholders based on their influence and interests, and to analyze their relationships using social network analysis. Examining these relationships can reveal areas of shared interest among stakeholders and provide a foundation for building collaborative structures that support effective implementation (21) of the proposed initiative to reduce the negative impacts of inappropriate donated medicines.

## 2 Materials and methods

### 2.1 Study setting

This study is part of the Gen-MED larger research project designed to identify and characterize stakeholders involved in the donated medicine cycle across six countries: Japan, Canada, the United Kingdom, Uganda, Rwanda, and Nigeria. Japan was chosen as the first country site due to its extensive experience in disaster management (26) and its role as a pharmaceutical hub with a high domestic response expertise (27). Stakeholders were defined as “*any individual or organization that can be directly or indirectly affected by, have an influence on, or have an interest in the development of a donated medicine tool/database in Japan*” [adapted from Franco-Trigo et al. (21)].

### 2.2 Study design

**Figure 1:**
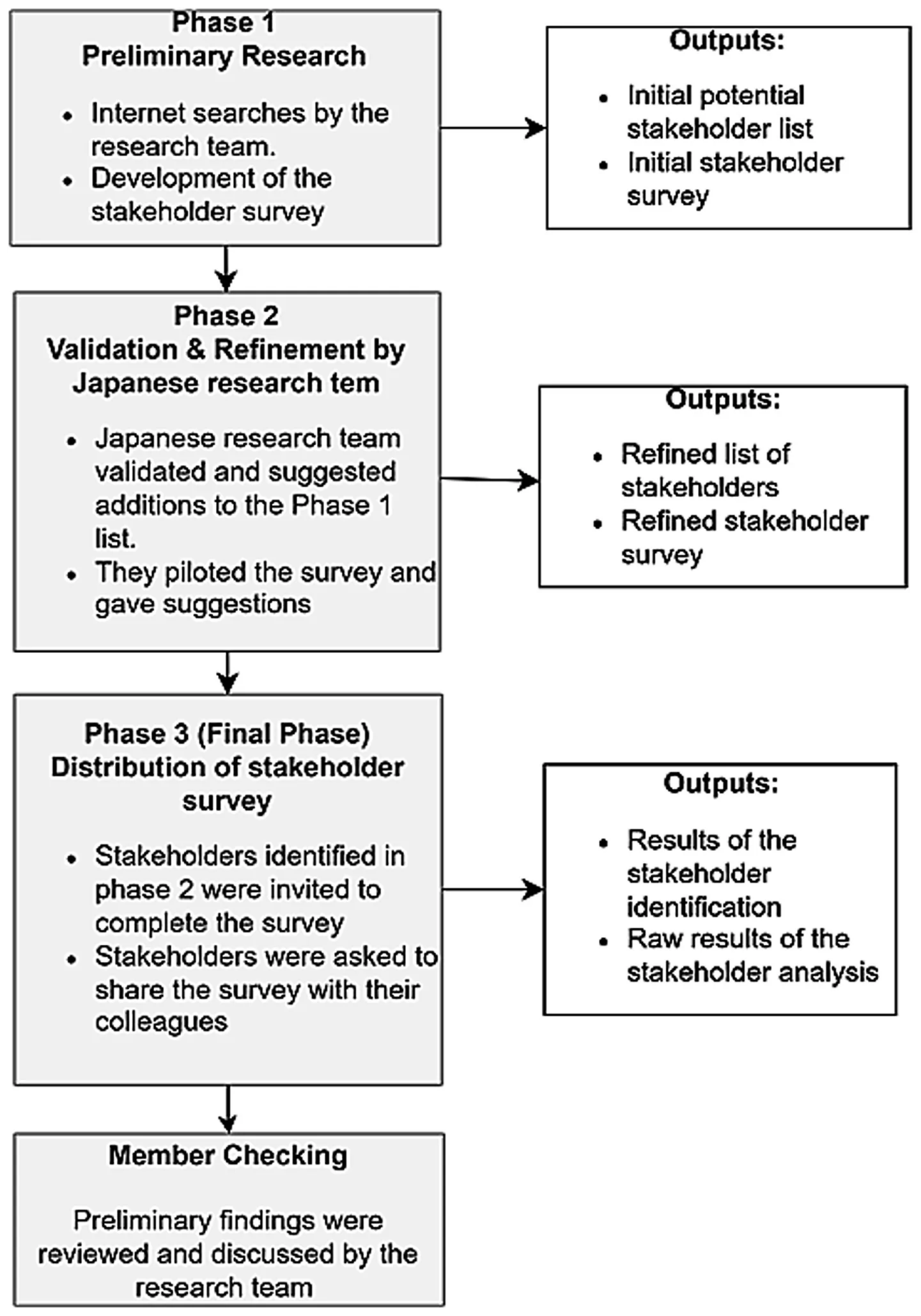
Study phases.

The stakeholder analysis was guided by the framework proposed by Gilmour and Beilin (22). It incorporated the three key activities for applying stakeholder analysis methods outlined by Reed et al. (23): (1) stakeholder identification; (2) stakeholder categorization/differentiation; and (3) analysis of stakeholder relationships. The procedures used to implement each of these activities, and the overall progression of the research process, are described below and illustrated in Figure 1.

#### 2.2.1 Stakeholder identification

The stakeholder identification process was conducted in three phases (see Figure 1) using purposive and snowball sampling (22) to ensure a comprehensive range of perspectives. First, the research team developed an initial list of stakeholders through internet searches and relevant literature, which also informed the design of the stakeholder questionnaire. The search was initially conducted by CN in English identifying Japan humanitarian organizations present on the internet. This list was further developed and refined by our Japanese research team members (E.K., K.T.M., T.I., and T.E.) who have expert knowledge of the disaster medicine system in Japan. This spanned government, academia, and humanitarian organization disaster medicine response and supply chain. Furthermore, participants were given the opportunity to identify missing stakeholders and share the survey within their networks. The final stakeholder list was compiled based on these responses and used for analysis.

#### 2.2.2 Stakeholder categorization/differentiation

Following stakeholder identification, representatives from the identified organizations were invited to complete a structured questionnaire. Participants received an overview of the proposed initiative and the stakeholder list. Drawing on the framework by Gilmour and Beilin (22), the questionnaire assessed each organization’s attributes, level of interest, involvement, and concerns (see Supplementary File 1 for the full questionnaire). The attribute definitions were adapted from Franco-Trigo et al. (21) and were as follows:

- Influence: “the current capacity of your organization to influence the initiative. It includes the access, availability and mobilization of resources; and/or the ability to mobilize other stakeholders and their resources; and/or the power to carry out potential activities or projects.” Response options: none, low, medium, high, and unsure.
- Attitude: “the current readiness of your organization towards the aforementioned initiative.” Response options: none, low, medium, high, and unsure.
- Interest: “level of interest that your organization currently has over the aforementioned initiative.” Response options: none, low, medium, high, and unsure.

#### 2.2.3 Analysis of stakeholder relationships

This phase examined collaborative relationships among the identified organizations by assessing reported engagement with other stakeholders on the list over the preceding two-year period (2023–2024). This timeframe was selected to ensure high recall accuracy and allowed gathering useful information. The intention behind this question was to check if a collaboration network among the identified organizations already existed and, if so, see which organizations stood out in the network. We operated under the assumption that if a network existed, the stakeholders that stood out in the existing collaboration network would probably be key to building the collaborative initiative and their experience and willingness to collaborate with other stakeholders would be necessary to make it successful. Different levels of collaboration were defined as per Franco-Trigo et al. (21) as follows and the list of stakeholders was provided again to facilitate responses:

- Not linked (do not work together).
- Communication (share information only).
- Cooperation (work together to achieve common goals).
- Collaboration (work together as a formal team with specific responsibilities).
- Fully linked (work together as a formal team; mutually plan and share staff or resources to accomplish goals).

### 2.3 Selection and recruitment of participants

The Japanese research team members (E.K., K.T.M., T.I., and T.E.) have extensive expertise in pharmaceutical systems and/ are involved in the donated medicine system in Japan. They provided forward and back translation of the study materials and supported ethics approval (ID: 3184) from Kumamoto University Research Ethics Committee. The Japanese research team members distributed the questionnaire by email to their contacts from the identified stakeholder list on behalf of the research team, using personalized links. One representative per organization was initially invited to participate or nominate an appropriate respondent. The participants were invited, in the questionnaire, to share a generic link with any stakeholder organizations not mentioned within the stakeholder list that they believed were involved in the donated medicine system, and/or share it with their colleagues within their organization (if appropriate). This resulted in an additional two stakeholders, taking the final stakeholder list to 19. Non-respondents received up to four reminders over a three-month period.

### 2.4 Data collection methods

To collect information from stakeholders, an online web-based questionnaire was developed using Qualtrics survey software (Qualtrics, Provo, UT) and was informed by the stakeholder analysis framework proposed by Gilmour and Beilin (22). The survey was available in both English and Japanese, following a forward–back translation process conducted by NA and TI and integrated into the Qualtrics platform. The forward and back translation was used as a quality control process where the study materials were translated into a Japanese, then a second team member translates it back to the original language to check accuracy. Key definitions were embedded within the questionnaire. The questionnaire was piloted by the research team (see Supplementary File 1). The questionnaire was included in the invitation emails (written in Japanese) and were distributed by the Japanese research team members. The email also included instructions on how participants could switch between language versions of the survey within the Qualtrics platform from English to Japanese. Data collection was conducted between June and August 2025.

### 2.5 Data analysis

The data analysis was primarily conducted by one author (CN), and reviewed by the team. Data on stakeholder attributes (i.e., level of influence, interest, and attitude) were analyzed by plotting influence against interest in a square matrix. Stakeholders were assigned to specific cells based on their responses. Using the classification proposed by Gilmour and Beilin (22), stakeholders were grouped as: (a) players—those with medium to high influence and interest; (b) context-setters—those with medium to high influence but low or no interest; (c) subjects—those with low or no influence but medium to high interest; and (d) crowd—those with low or no influence and interest. Stakeholder attitudes were indicated within the matrix using color coding (green = high, black = medium, red = low, yellow = unsure, blue = none).

A descriptive analysis was conducted for questions related to stakeholders’ specific interests in the initiative, their desired level of involvement, and the identification of key stakeholders. Questions related to stakeholder relationships (i.e., level of collaboration) were analyzed using social network analysis. A whole-network analysis was conducted that included all identified stakeholders, as well as additional organizations that participated using the anonymous survey link. Data were entered into a square matrix and analyzed using UCINET 6.813 (28), with network visualizations generated in NetDraw 2.195 (29). Since the relationships examined were reciprocal, tie values were symmetrized; that is, the relationship between stakeholders A and B was treated as identical to the relationship between B and A (30). For each pair of stakeholders (dyad), the relationship value was defined using the higher score reported by either party (21, 31). If a stakeholder did not participate in the study, or participated but did not respond to this question, the score provided by the other stakeholder was used to determine the relationship value. Similarly, if a stakeholder participated in the study but was not on the initial list, the value they score other stakeholders were used to define the relationship value (21). Data were not dichotomized, instead, ties were treated as valued networks to capture the varying strength of connections among stakeholders. This avoids the oversimplification of treating relationships as binary, thereby preserving the importance of differing levels of engagement among stakeholders (32).

Several measures were calculated to assess the properties of the network as a whole. To assess how well-connected the network was Borgatti et al., the following measures were examined: number of ties: the total number of ties in the network; density: number of existing ties out of all the possible ties in the network; and average degree: “average number of ties” that stakeholders had Borgatti et al. (30). To examine the overall structure of the network (21), the following measures were assessed: centralization: the extent to which the network is organized around one stakeholder or a group of stakeholders; and core–periphery structure: the extent to which the network reflects a structure composed of two groups, core stakeholders, who are highly connected to one another and to others in the network, and periphery stakeholders, who are primarily connected to core stakeholders but not to each other. Additional measures were calculated to understand the properties of the network at the stakeholder or node level (21, 33). These include; degree centrality: the actual number of ties a stakeholder has, used to identify the most important or powerful stakeholders in the network; and betweenness centrality: the frequency with which a stakeholder lies on the shortest path between two other stakeholders, used to identify strategic stakeholders who may control the flow of information or resources within the network.

For this study, critical stakeholders were determined by triangulating multiple sources of data. This triangulation improved the thoroughness of the analysis, enhancing both the quality of the findings and confidence in the results (21, 34). The following criteria were used to identify critical stakeholders: (a) stakeholders who reported high levels of influence and interest; (b) the first quartile of stakeholders with most votes considered key by the other stakeholders; (c) the first quartile of stakeholders with higher degree centrality in the collaboration network; and (d) the first quartile of stakeholders with higher betweenness centrality in the collaboration network. This ensured that all four methods contributed to a similar extent to the selection of critical stakeholders.

### 2.6 Member checking

The preliminary findings were reviewed and discussed by the research team to ensure interpretive consistency. During this process, one of the initially identified stakeholders was removed because it became clear from their questionnaire response that their operational scope fell outside the medicine donation system being studied and therefore were ineligible to complete the study (changing the final list of stakeholders from 19 to 18 organizations). Integrating the perspectives of the broader research team with the insights provided by the Japanese research team strengthened the trustworthiness of the findings (34), as it was grounded in contextual knowledge of the disaster management system in Japan. This was specifically designed to mitigate individual investigator bias and provide a more comprehensive stakeholder analysis (21).

### 2.7 Ethics statement

This research was the result of collaboration by three universities, ethical approval was obtained from the University of Alberta Human Research Ethics Committee (ID: Pro00146666), University of Nottingham School of Pharmacy Research Ethics Committee (ID: 001-2025), and Kumamoto University Research Ethics Committee (ID: 3184). Informed consent was obtained within the questionnaire with the first page including the information sheet. For privacy considerations, stakeholder names were de-identified, and only stakeholder groupings are reported in the paper.

## 3 Results

Of the 18 organizations, 16 representatives from 15 organizations (83% organizational response rate) agreed to participate and completed the survey. Participants were, on average, 55.3 years old (SD = 9.04; range: 36–68). Gender representation was heavily skewed towards males, accounting for 13 individuals, while females constituted only 3 participants, as illustrated in Table 1.

**Table 1:** Participant characteristics.

Characteristic	Category	Overall sample	Pharmacy professional bodies	Regional hospitals	Regional government	Humanitarian organization	University	Pharmaceutical Wholesaler	National pharmaceutical manufacturers’ association
Stakeholder sub-categories		16	5	4	2	1	2	1	1
Age	Mean	55	57	52	36	49	67	52	66
Gender	Male	13	5	4	2		1		1
	Female	3				1	1	1	
Roles of stakeholder representatives	Pharmacist	6	3	2				1	
	Professor	3		2			1		
	Supply logistics	3			2	1			
	Chairman	2	1				1		
	Director general	1							1
	Disaster prevention committee member	1	1						
Type of health emergencies involved with* N = 28	Natural hazards	13	4	3	2	1	1	1	1
	Long term disasters and emergencies	11	4	3	1	1	1		1
	Others (radiation exposure)	4		3			1		
Availability of policies on drug donation	Yes	3	1		1	1			
	No	9	3	3	1			1	1
	Unsure	4	1	1			2		
Type of donated medicines involved with* N = 28	Vaccines	4		1		1	1		1
	Acute care drugs	10	4	3		1	1		1
	Chronic condition drugs	7	2	3		1			1
	Others (e.g., medical supplies)	7	1	2	1	1	1	1	

* Stakeholders could select more than 1 category.

### 3.1 Stakeholder identification

**Figure 2:**
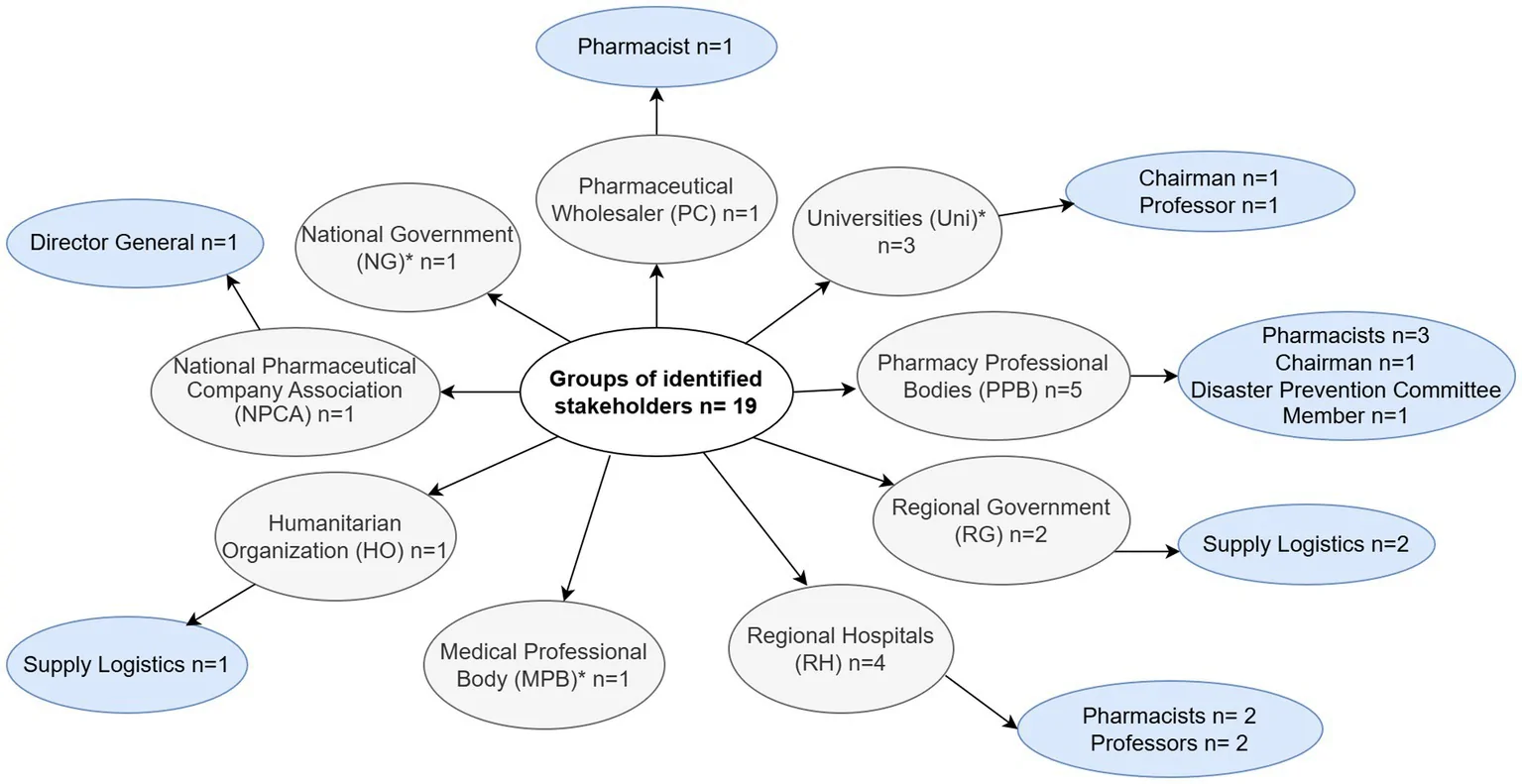
Identified stakeholder groups.

A total of 18 stakeholders were identified at the national and regional levels, belonging to 9 groups shown in the stakeholder map presented in Figure 2. (1) Pharmaceutical Wholesaler (PC, *n* = 1); (2) Regional Government (RG, *n* = 2); (3) Universities (Uni, *n* = 3); (4) Regional Hospitals (RH, *n* = 4); (5) Pharmacy Professional Bodies (PPB, *n* = 4); (6) not-for-profit Humanitarian Organization (HO, *n* = 1); (7) National Pharmaceutical Manufacturers’ Association (NPMA, *n* = 1); (8) National Government (NG, *n* = 1); and (9) Medical Professional Body (MPB, *n* = 1). Three stakeholder representatives from three groups (Uni, NG, and MPB) did not participate in the study. Eight stakeholders were non-government organizations, two were public, three were government, two not-for-profit, and one was a private organization.

### 3.2 Stakeholder categorization/differentiation

**Figure 3:**
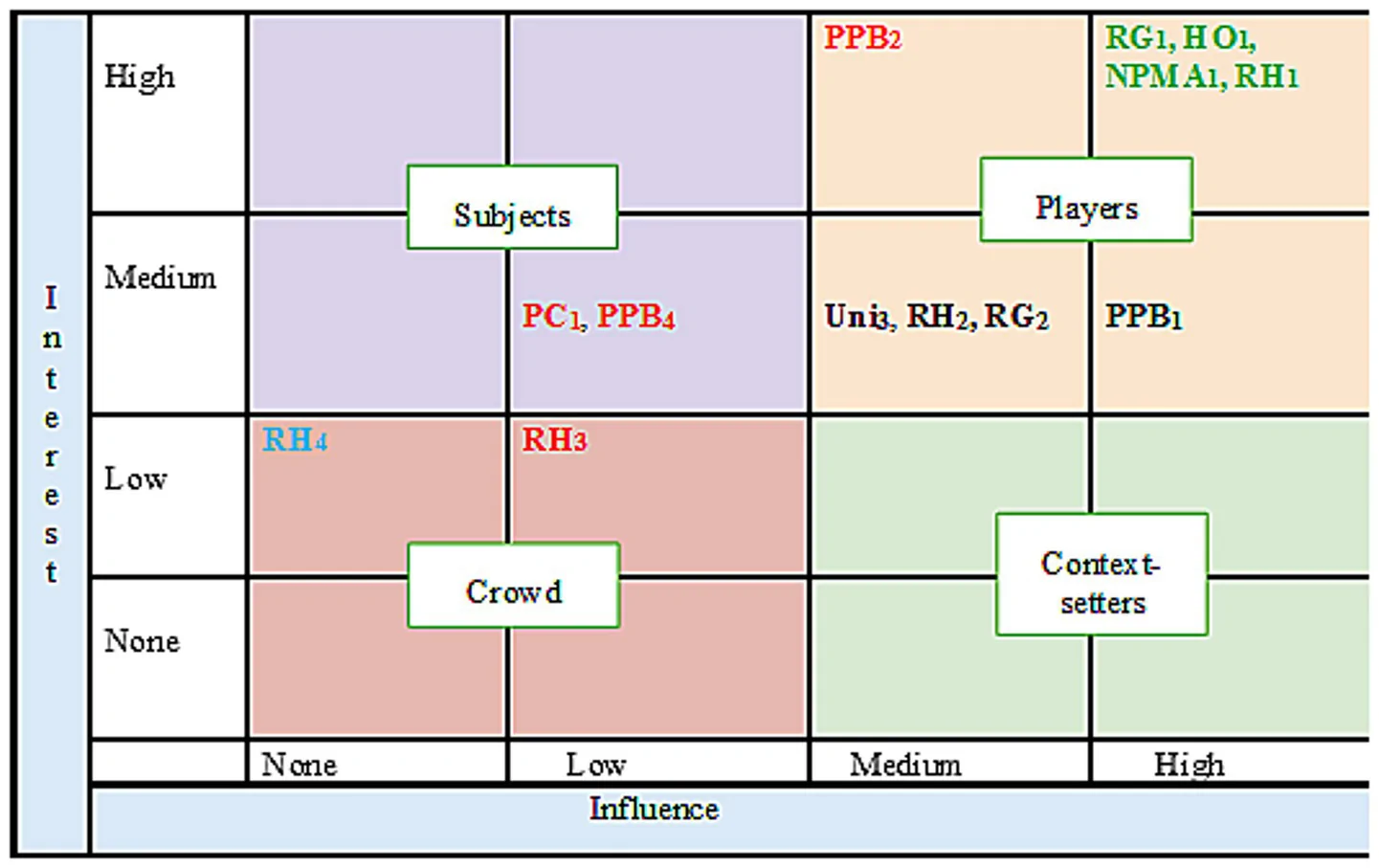
Stakeholder influence-interest matrix. This figure shows the influence, interest and attitude self-reported by stakeholders towards the initiative. PPB, Pharmacy Professional Body; RH, Regional Hospital; RG, Regional Government; HO, Humanitarian Organization; Uni, University; PC, Pharmaceutical wholesaler NPMA, National Pharmaceutical Manufacturers’ Association. Stakeholder attitudes toward the initiative were indicated using color coding (green = high, black = medium, red = low, yellow = unsure, blue = none).

Stakeholders’ self-perceived levels of influence and interest, along with their attitudes toward the initiative, are presented in the influence–interest matrix (Figure 3). The majority of participants were classified as players (*n* = 9). Public health was the most commonly reported area of interest, by 12 stakeholders across seven groups. This was followed by an interest in building relationships with other stakeholders (six stakeholders from four groups) and scientific interests (five stakeholders from four groups). Additionally, three stakeholders from two groups reported a political interest in the initiative, while two stakeholders from two groups indicated an economic interest in the collaborative initiative (see Supplementary Table 1). The stakeholders’ desired involvement in the initiative can also be found in the Supplementary Table 1. In addition, stakeholders expressed concerns over the initiative (see Table 2).

**Table 2:** Stakeholder concerns regarding the initiative.

Identified concerns of the proposed initiative
1. Participants questioned the ability to guarantee the quality, efficacy, and safety of donated medicines from a hygienic perspective.
2. Another concern was the lack of standards for donating medicines, including the view that products with expiration dates remain the property of the company until they expire.
3. Participants highlighted concerns related to leadership and management across their organizations, noting inconsistent structures and unclear roles and responsibilities.
4. Participants emphasized that needs vary depending on the type of disaster, and questioned whether the necessary medicines can be secured at the required time. They stressed the importance of creating separate systems for pre-disaster and disaster situations and whether such a system can be effectively established.
5. Reaching consensus with relevant parties was also identified as a concern.
6. Participants noted that general staff have little knowledge about disasters, demonstrate low levels of interest and lack actual experience.
7. Lack of a system for cooperation was also a concern.

### 3.3 Stakeholder relationships

**Figure 4:**
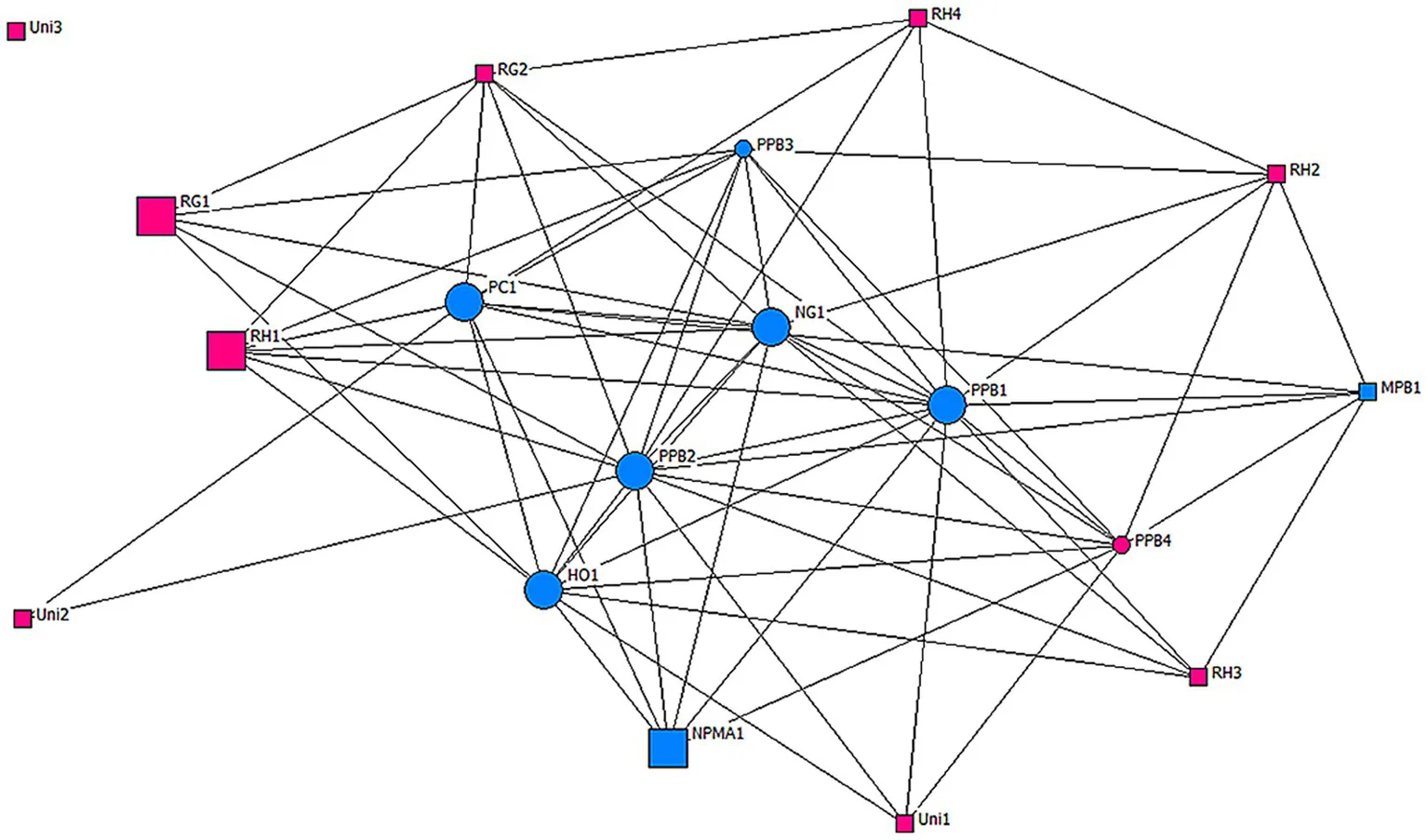
Collaboration network among stakeholders. This figure shows the collaboration network that exists among the stakeholders. One stakeholder (Uni3) was not connected to other stakeholders in the network. The blue color represents the national stakeholders and the pink color represents the regional stakeholders. The circles represent the core stakeholders, the squares represent the periphery stakeholders. The bigger circles/squares represent critical stakeholders.

Social network analysis results revealed an existing collaboration network among 17 of the 18 stakeholder organizations, with a total tie weight of 262. All stakeholders, except one (Uni_3_), were connected within the network, meaning that each could reach any other stakeholder through existing collaboration ties. The density of this network was 0.885, and the average weighted degree was 14. The network centralization was 25.55%, and its structure was moderately correlated with a core/periphery structure (correlation = 0.73). That is, there was a group of stakeholders with high links among them (core), and another group of stakeholders linked to the stakeholders in the core group but poor or no links between them (periphery). Figure 4 shows the core/periphery structure of the network displaying the stakeholder groups and critical stakeholders at the national and regional levels.

### 3.4 Critical stakeholders: combination of results

To systematically identify critical stakeholders, this study combined four approaches. The first two approaches were used for stakeholder differentiation and categorization: *(1) stakeholders who reported high levels of self-perceived influence and interest* (see Table 3, Column A), and *(2) stakeholders identified as key actors by other stakeholders* (see Table 3, Column B). Stakeholders identified as key represented four distinct groups: a pharmacy professional body (*n* = 1), a national government entity (*n* = 1), a humanitarian organization (*n* = 1), and a national pharmaceutical manufacturers’ association (*n* = 1). To protect the identity of individual stakeholders, respondents’ explanations for why these stakeholders were considered key were synthesized and reported by stakeholder groups. A summary of these reasons is presented in Supplementary File 2.

**Table 3:** Critical stakeholders.

Stakeholder groups	A	B	C	D
	High influence and interest (self-perception)	1st quartile voted as key stakeholders (number of votes)	1st quartile higher degree centrality [more power] (normalized degree centrality value)	1st quartile betweenness centrality [strategic] (normalized betweenness centrality value)
PPB		**PPB**_**1**_ **(12)**	**PPB**_**1**_ **(44.1)** **PPB**_**2**_ **(38.2)**	**PPB**_**1**_ **(9.6)** **PPB**_**2**_ **(16.0)**
NG		**NG**_**1**_ **(15)**	**NG**_**1**_ **(38.2)**	**NG**_**1**_ **(5.5)**
RG	RG_1_			
HO	**HO**_**1**_	**HO**_**1**_ **(11)**	**HO**_**1**_ **(38.2)**	**HO**_**1**_ **(4.4)**
NPMA	**NPMA**_**1**_	**NPMA**_**1**_ **(9)**		
RH	RH_1_	–	–	–
PC	–	–	**PC**_**1**_ **(32.4)**	**PC**_**1**_ **(6.6)**

PPB, Pharmacy Professional Body; NG, National Government; RG, Regional Government; HO, Humanitarian Organization; NPMA, National Pharmaceutical Manufacturers’ Association; RH, Regional Hospital; PC, Pharmaceutical Wholesaler. In bold are national stakeholders; and in black are regional stakeholders.

The third and fourth approaches were derived from the social network analysis of stakeholder relationships: *(3) degree centrality within the collaboration network* (see Table 3, Column C), and *(4) betweenness centrality within the collaboration network* (see Table 3, Column D). Integrating results from these four methods led to the identification of eight critical stakeholders. As shown in Table 3, Column C, identifying the first quartile of stakeholders with the highest degree centrality (i.e., those more linked to other stakeholders) led to the selection of five stakeholders pertaining to the following groups: pharmacy professional bodies (*n* = 2); national government (*n* = 1); humanitarian organization (*n* = 1); and pharmaceutical wholesaler (*n* = 2). Similarly, the first quartile of stakeholders with the highest betweenness centrality (i.e., those in the path that links two other stakeholders) was constituted by five critical stakeholders (see Table 3, Column D), belonging to the same groups. Considering the degree centrality and betweenness centrality together, five stakeholders were both well connected to others and strategically situated in the network.

Including all four methods, eight critical stakeholders were identified from pharmacy professional bodies, regional and national government, humanitarian organizations, the national pharmaceutical manufacturers’ association, regional hospitals, and pharmaceutical companies. Only one stakeholder, a humanitarian organization was identified as critical by all four methods. Two additional stakeholders, a pharmacy professional body and a national government actor were identified as critical by three of the four methods. Overall, the results indicate that no critical stakeholders were identified within two of the nine stakeholder groups: medical professional bodies and universities. It should be noted that only two stakeholders from the regional level, a regional hospital and government were identified as key stakeholders. This identification was made by only one method, based on self-perceived high levels of interest and influence. Furthermore, a pharmaceutical wholesaler stakeholder with a low attitude for the initiative was identified as a critical stakeholder by two methods: degree centrality and betweenness centrality measures.

## 4 Discussion

This study demonstrates how integrating stakeholder analysis with social network analysis does not only identify influential actors but also reveal the underlying patterns of collaboration that exist in the current Japanese donated medicines system. While our findings demonstrate a substantial willingness among stakeholders to collaborate, the successful implementation of the proposed initiative requires a structure that recognizes their shared interests and differing concerns related to the project. These insights provide an empirical basis for establishing an appropriate steering group and managing a collaborative process that balances the diverse needs, interests, and perspectives of the stakeholders involved (21). Notably, the positioning of half of the critical stakeholders as players within the influence–interest matrix, suggests that the proposed initiative is supported by actors who not only perceived themselves as powerful but also demonstrate a high interest in its implementation. This is important because it may increase the feasibility of the collaborative approach. By triangulating these results with the key stakeholders identified as key by other stakeholders, the proposed initiative gains the legitimacy necessary for the sustainable collaborative process (21). Furthermore, the centrality measures identified the most connected and strategic stakeholders in controlling the flow of resources and information through the network. Knowing these critical stakeholder groups allows for prioritizing stakeholders for engagement purposes in planning and implementation of the proposed initiative. Additionally, the network visualization may assist in the design of communication strategies to raise awareness of this particular initiative, as well as in the design of future mitigation strategies to address the environmental, economic, social, and health impacts of donated medicines (21).

National level organizations including the government, humanitarian organizations, and pharmacy professional associations emerged as critical stakeholders and the most connected within the donated medicines system. Their prominence within the collaboration network reflects the central role that national-level actors play in providing coordination capacity and resources necessary for establishing and implementing large-scale initiatives in disasters and humanitarian crises (21). As noted by Varvasovszky and Brugha, the delegation of responsibilities and allocation of financial resources to national institutions can create the conditions to advance strategic policy goals (25). In Japan, these national stakeholders are pivotal in coordinating emergency health responses, overseeing medical services, pharmaceutical regulation, health financing, and broader health system planning. This enables the effective management of medicine supply, demand, and cost control during crises (35). This coordination is often mirrored by national humanitarian organizations, which work with the government to manage the distribution of donated medicines (36), and pharmacy professional bodies, which oversee the deployment of pharmacists, provision of pharmaceutical care, and medication dispensing during emergencies (37). However, while national-level actors provide the necessary regulatory and financial framework (35), the success of the proposed initiative depends on a governance structure that effectively integrates regional stakeholders. Despite representing a smaller proportion of the critical actors, regional stakeholders serve as a crucial link between national policy and local implementation. Given Japan’s highly decentralized public health system (38), these actors are essential for the coordination and implementation of health-related activities at regional and local levels (38, 39). Because these actors possess contextual knowledge of local needs and implementation challenges, their early involvement is essential to ensure that the proposed initiative is both feasible and operationally effective (21).

Universities and medical professional bodies were not identified in this study as one of the eight critical stakeholders, potentially reflecting their indirect involvement in the operational management of donated medicines. More research into their indirect involvement of donated medicines may be warranted as some of these organizations like pharmacy schools in Japan house the mobile pharmacies used in disaster response, and contributing to the volunteer workforce to respond to pharmaceutical needs in a disaster (14). However, as demonstrated by Aoki and Ito, academic institutions in Japan are often perceived primarily as knowledge producers rather than frontline implementers during emergencies (40). While these institutions possess significant resources including specialized expertise, research infrastructure, and technical innovation (40), their direct role in donated medicine logistics remains limited. Similarly, the primary mandate of medical professional bodies is centered on clinical care delivery. During emergencies, their resources are prioritized for the deployment of medical assistance teams to provide immediate patient support (41). Consequently, while these actors may not be critical in the donated medicines system, they remain vital partners for the other aspects of pharmacy disaster response as it may relate to the proposed initiative. A notable finding in the stakeholder network was the position of pharmaceutical companies, which exhibited high structural connectivity alongside a relatively low attitude toward the proposed initiative. While these companies are central to the medicine production and supply chains (42), their hesitation may be influenced by concerns related to regulatory compliance and the operational complexities associated with donated medicines programs. For example, findings of Flores and colleagues suggest gaps between pharmaceutical companies’ practices and compliance with the WHO guidelines for donated medicines (43), noting that only 15% of pharmaceutical companies involved with donated medicines have publicly available donation policies, highlighting limited transparency and accountability in donation practices. Despite these concerns, pharmaceutical companies have historically mobilized significant resources including the provision of medicines following major disasters in Japan (44). Therefore, targeted engagement with the private sector is a strategic necessity to foster more positive support and ensure the feasibility and long-term sustainability of the proposed initiative.

### 4.1 Next steps

Findings from this stakeholder and social network analysis have already been shared with partners in Japan through member checking and they provided contextual validation and interpretation of the results. Building on this, the next phase of the Gen-MED project will extend similar analyses to the remaining countries (Canada, the United Kingdom, Uganda, Rwanda, and Nigeria) to enable cross-country comparison of stakeholder dynamics and collaboration patterns in the donated medicines system. While Japan served as the initial country, the structural insights generated from this study provide a transferable methodological approach that can be applied in other countries and humanitarian settings to identify and engage critical stakeholders when developing strategies to mitigate the negative impacts of inappropriate donated medicines. Future research could include an in-depth exploration of the Japan’s donated medicines lifecycle. Similar to ongoing work in Uganda, qualitative methods such as stakeholder interviews could be used to better understand processes, challenges, and decision-making across the donated medicines system, complementing the findings from the current stakeholder analysis.

### 4.2 Strengths and limitations

This study provides a significant methodological contribution by incorporating social network analysis into the stakeholder analysis framework. This approach enabled the mapping of the existing collaboration network within the Japanese donated medicines system and provided a deeper understanding of stakeholder influence and interest (45). The use of a self-administered questionnaire allowed for the inclusion of diverse stakeholders across broad geographical areas. Furthermore, the study design was strengthened by the involvement of the local researchers in Japan. The triangulation of four distinct methods to identify critical stakeholders ensures the robustness, quality, and credibility of the findings (34). While the results are context-specific, the identified stakeholder groups and the selection criteria used may serve as a useful framework for fast-tracking stakeholder identification in other settings. Some limitations should be acknowledged in the interpretation of these results. First, three invited stakeholder groups did not participate. Second, one stakeholder organization was represented by two individuals whose responses differed. While this internal variance is noted, having multiple representatives from a single organization can be advantageous, as it captures a broader range of internal perspectives and reduces the risk of individual bias. Third, potential language barriers may have influenced responses, however, this was mitigated by providing the questionnaire in both English and Japanese using a forward–back translation process and locally distributed invitations in Japan. Finally, as with any stakeholder analysis, these results are context-specific and temporary. This is because stakeholder attributes, interests, and influence may shift due to changes in the economic, political, or social landscape, or as the initiative evolves through different planning phases (21). Additionally, the use of retrospective (2023–2024) collaboration data to identify existing relationship to infer future stakeholder roles has limited predictive validity.

## 5 Conclusion

This stakeholder analysis provided a comprehensive understanding of the patterns of collaboration within the Japanese donated medicines system and offers important insights into the context required for the successful implementation of the proposed initiative. A diverse group of critical stakeholders, mostly national-level actors were identified, whose positions within the network indicated their capacity to influence decision-making processes, mobilize resources, and facilitate the flow of information across the network. These insights can inform future guidance for designing collaborative approaches that can enhance transparency and better align donated medicine practices with local health needs while supporting compliance with the WHO guidelines for donated medicines.

## Data Availability

The original contributions presented in the study are included in the article/Supplementary material, further inquiries can be directed to the corresponding author.
