# Supplementary material for: Navigating the Generosity of Medicines (Gen-MED): characterizing the complex relationships of medication donations in Japan through stakeholder and social network analysis
Source: Front Public Health. 2026 Aug 24;14:1866926. doi: 10.3389/fpubh.2026.1866926 (PMC13547525; doi:10.3389/fpubh.2026.1866926)
Supplement: Supplementary file 1 [file Supplementary_file_1.docx]

Supplementary File 1. Full Questionnaire

***Questions were derived from the stakeholder analysis framework proposed by Gilmour and Beilin.^1^***

Q1 What is the Organization that you are affiliated with?   *[Note: this information will not be reported to anyone or used in the published results. It will only be accessed by the researchers for recruitment purposes]*

___________________________________________________________

 Q2 What is your role in the Organization?

________________________________________________________________

Q3 What types of health emergencies are your organization involved with? *[Please select all the apply]*

▢ Natural Hazards *(e.g., floods, earthquakes, fires, etc.)*  (1)

▢ Long-term disasters and emergencies *(e.g., conflicts, health system support/establishment)*  (2)

▢ Other (please specify):  (3) __________________________________________________

Q4 Which of the following best describes how your organization is involved with regard to donated medicines (e.g., being involved, managing, or coordinating)? *[Please select all the apply]*

▢ Exportation  (1)

▢ Importation  (2)

▢ Inventory of donated medicines  (3)

▢ Disposal of the expired/unused medicines  (4)

▢ Other (please specify):  (5) __________________________________________________

Q5 Does your country or organization have any policies on drug donations?

o Yes. If so, please provide a publicly available link  (1) __________________________________________________

o No  (2)

o Unsure  (3)

Q6 What type of donations of medicines does your organization involved with? *[Include both prescriptions and over-the-counter, Please select all the apply]*

▢ Vaccines  (1)

▢ Acute care drugs  (2)

▢ Chronic condition drugs  (3)

▢ Other (please specify):  (4) __________________________________________________

Q7 Which sex do you identify as?

o Male  (1)

o Female  (2)

o Prefer not to say  (3)

Q8 What is your age?

________________________________________________________________

Q9 What is your email address?  [We may use this email address for administration purposes to confirm your response. We will de-identify your personal information from data collected.]

________________________________________________________________

Q10 From the following list, please indicate which stakeholders do you consider ***'key' stakeholders (i.e., absolutely essential)*** to carry out the initiative mentioned above.	* For this study we consider that you know a stakeholder if you have enough information on them to issue an opinion. We define a key stakeholder as "*“any individual or organization that can be directly or indirectly affected by, have an influence on, or have an interest in the development of a donated medicine tool/database”*

| List of stakeholders | I do not know this stakeholder (1) | Key stakeholder (please specify reasons why you think this in the text box below the stakeholder name) (2) | Not a key stakeholder (please specify reasons why you think this in the text box below the stakeholder name) (3) |
| --- | --- | --- | --- |
|  | o | o | o |

Q11 Please write below any other stakeholders that should be included that are not mentioned above. *[Would you be willing to share the survey with them on our behalf, or making the introduction? We will email you a unique survey link to share with them. If not, the research team will contact a main contact email address of the organisation you stated below, without mentioning your involvement/participation.]*

Q12 On the following list, please indicate which stakeholders your organization collaborated with in 2023 and 2024  Definitions ***Not linked:*** do not work together ***Communication:*** share information only ***Cooperation:*** work together to achieve common goals ***Collaboration:*** work together as a formal team with specific responsibilities ***Fully linked:*** work together as a formal team; mutually plan and share staff or resources to accomplish goals

| List of stakeholders | Not Linked (1) | Communication (2) | Cooperation (3) | Collaboration (4) | Fully Linked (5) | Do not Know (6) |
| --- | --- | --- | --- | --- | --- | --- |
|  |  |  |  |  |  |  |

Q13 For the below questions, please keep the following in mind.   - All information is confidential and no names, organizations, or identifying information will be published  - We are referring to the process of managing donated medicines sent/received throughout the donated medicine lifecycle  - The initiative we are proposing is building transparency into the system, increasing the proportion of donated medicines that meet the health needs of the region following health emergencies and complies with the World Health Organization’s guidelines

Q14 How would you classify the interests of your organization would have regarding this initiative? *[Please select all the apply]*

▢ Politic  (1)

▢ Financial/Economic  (2)

▢ Scientific/Technical  (3)

▢ Public health/General interest/Social aspects  (4)

▢ Building relationships/Access to other stakeholders  (5)

▢ Other: Please specify  (6) __________________________________________________

Q15 To what extent do you think your organization would like to be involved in the initiative? *[Please select all the apply]*

▢ Would not like to be involved  (1)

▢ Only receiving information  (2)

▢ Be a consultative body  (3)

▢ Active participation in the process without making decisions  (4)

▢ Active participation in the process and in decision-making  (5)

▢ Other: Please specify  (6) __________________________________________________

Q16 What do you think would be the biggest concern for your organization in relation to an initiative like the one mentioned above?

________________________________________________________________

________________________________________________________________

Q17   In the below table, please select the option that best describes your perspective on how your organization would be positioned with respect to a project as the one described above, in terms of:	** If you are unsure, please save the survey and go and ask your colleagues, or please forward the survey link to them to also complete and provide some information.*

|  | **None** (1) | **Low** (2) | **Medium** (3) | **High** (4) | **Unsure** (5) |
| --- | --- | --- | --- | --- | --- |
| ***Influence:*** the current capacity of your organization to influence the initiative. It includes the access, availability and mobilization of resources; and/or the ability to mobilize other stakeholders and their resources; and/or the power to carry out potential activities or projects (1) | o | o | o | o | o |
| ***Attitude:*** the current readiness of your organization towards the aforementioned initiative (2) | o | o | o | o | o |
| ***Interest:*** level of interest that your organization currently has over the aforementioned initiative (3) | o | o | o | o | o |

Q18 Would you like to make any additional comments to the above questions about interest, attitude, and influence?

________________________________________________________________

________________________________________________________________

Q19 Do you have any additional comments or information to provide about key stakeholders?
