# Supplementary material for: Navigating the Generosity of Medicines (Gen-MED): characterizing the complex relationships of medication donations in Japan through stakeholder and social network analysis
Source: Front Public Health. 2026 Aug 24;14:1866926. doi: 10.3389/fpubh.2026.1866926 (PMC13547525; doi:10.3389/fpubh.2026.1866926)
Supplement: Supplementary file 2 [file Supplementary_file_2.docx]

Supplementary File 2. Reasons why participants considered stakeholders “key stakeholders.”

The reasons stated by participants for considering particular stakeholders as key could be mainly organized as follows:

1. *Regulatory authority and legal mandate*, particularly for the national government, which has jurisdiction over pharmaceuticals, oversees compliance with relevant legislation (e.g., pharmaceuticals and medical devices regulations), ensures transparency in distribution, influences the specifications and operational structure of tools and databases related to donated medicines, and regulates the import and export of narcotics and psychotropic substances.
2. *Decision-making power in disaster response*, especially regarding the evaluation of the quality, efficacy, and safety of pharmaceuticals donated from overseas, as well as decisions concerning the acceptance and distribution of donated medicines and the development of national policies and cooperation mechanisms. This reason was primarily attributed to the national government.
3. *Technical and contextual knowledge,* including understanding disaster situations and medical needs, knowledge of pharmaceutical exports, and awareness of the quality, efficacy, and safety considerations related to donated medicines. This reason applied particularly to the national government and pharmacy professional bodies.
4. *Professional expertise and frontline experience,* particularly among pharmacists represented by the pharmacy professional bodies. Participants highlighted pharmacists’ roles as on-site users of donated medicines, their experience in dispatching pharmacists and operating temporary pharmacies during disasters, and their function as medicine suppliers, while noting that hospital pharmacists primarily support hospital practice and may not always be directly involved in medicine supply.
5. *Organizational networks and information dissemination capacity*, including the ability of the pharmacy professional bodies to compile opinions from other pharmacists’ associations and provide information to hospitals and clinics.
6. *Institutional experience and operational capacity*, particularly within the humanitarian organization. This was recognized for its disaster response experience, pharmacist networks within the organization, and perceived role in the management and supply of donated medicines.
