# Supplementary material for: Navigating the Generosity of Medicines (Gen-MED): characterizing the complex relationships of medication donations in Japan through stakeholder and social network analysis
Source: Front Public Health. 2026 Aug 24;14:1866926. doi: 10.3389/fpubh.2026.1866926 (PMC13547525; doi:10.3389/fpubh.2026.1866926)
Supplement: Supplementary file 3 [file Table_1.docx]

Supplementary Table 1. Stakeholders’ interests and level of involvement

|  |  |  | **Stakeholder categories** | | | | | | |
| --- | --- | --- | --- | --- | --- | --- | --- | --- | --- |
| Characteristic | Category | Total number of stakeholders (%) | PPB | RH | RG | HO | Uni | PC | NPMA |
| *Interests (N=31) | Politic | 3 (9.7) | 2 |  |  |  |  |  | 1 |
|  | Financial/Economic | 2 (6.5) |  | 1 |  |  |  |  | 1 |
|  | Scientific/Technical | 5 (16.1) | 1 | 1 |  |  | 2 |  | 1 |
|  | Public health/General interest/Social aspects | 12 (38.7) | 4 | 1 | 2 | 1 | 2 | 1 | 1 |
|  | Building relationships/Access to other stakeholders | 6 (19.3) | 2 | 1 | 2 | 1 |  |  |  |
|  | Others (emergency care, international relations, quality assurance, supply logistics) | 3 (9.7) |  | 1 | 1 | 1 |  |  |  |
| *Involvement in the Initiative (N=17) | Would not like to be involved | 0 |  |  |  |  |  |  |  |
|  | Only receiving information | 5 (29.4) | 2 | 2 |  |  | 1 |  |  |
|  | Be a consultative body | 1 (5.9) |  | 1 |  |  |  |  |  |
|  | Active participation in the process without making decisions | 5 (29.4) | 1 | 1 |  | 1 | 1 | 1 |  |
|  | Active participation in the process and in decision-making | 5 (29.4) | 1 | 2 | 2 |  |  |  |  |
|  | Others (involvement limited to pharmaceutical-related aspects) | 1 (5.9) |  | 1 |  |  |  |  |  |

***PPB****: Pharmacy Professional Body;* ***RH****: Regional Hospital;* ***RG:*** *Regional Government;* ***HO****: Humanitarian Organization;* ***Uni:*** *University;* ***PC****: Pharmaceutical Wholesaler;* ***NPMA****: National Pharmaceutical Manufacturers’ Association. *Stakeholders could select more than one category*
